# Anaerobic metabolism during short all-out efforts in tethered running: Comparison of energy expenditure and mechanical parameters between different sprint durations for testing

**DOI:** 10.1371/journal.pone.0179378

**Published:** 2017-06-09

**Authors:** Filipe Antônio Barros Sousa, Rubens Eduardo Vasque, Claudio Alexandre Gobatto

**Affiliations:** 1 School of Applied Sciences, University of Campinas, Limeira, Sao Paulo, Brazil; 2 Einstein Integrated Faculties of Limeira, Limeira, Brazil; University e-Campus, ITALY

## Abstract

This study’s aims to verify the energy expenditure, metabolic distress and usefulness to evaluate the anaerobic constructs for different all-out durations in running efforts. Twelve active male underwent four testing sessions, one for familiarization and three performing one all-out (AO) tethered running sprint lasting 30s, 20s or 10s. Oxygen consumption, excess post exercise oxygen consumption, and lactate production were retained to analyse metabolic function, together with mechanical power and work as performance parameters. Paired results were compared via one-way ANOVA for repeated measures (Tukey-HSD post-hoc), effect sizes and ICC for absolute agreement. Statistical significance was accepted at p ≤ 0.05. Despite total and energy expenditure from oxidative pathway being significantly higher for longer durations (p < 0.001; ES > 0.7), glycolytic energy expenditure presented an agreement between AO30s and AO20s (ICC-A = 0.63*), while the paired comparisons to AO10s have presented significant differences (p < 0.01; ES > 1.0). Phosphagen energy expenditure were similar between all-out durations (p = 0.12; ICC-A = 0.62*; ES < 0.5). Maximum mechanical power was higher in AO10s than in AO30s (p = 0.03; ES = 0.6), not being different between AO10s and AO20s (p = 0.67; ICC-A = 0.88*; ES = 0.2) and between AO20s and AO30s (p = 0.18; ICC-A = 0.56*; ES = 0.4). In addition, agreement between work in the first ten seconds was confirmed via ICC only between AO10s and AO20s (p = 0.50; ICC-A = 0.86*; ES = 0.3), but not for the other paired comparisons (p < 0.1; ICC < 0.45; ES > 0.5). AO20s is a better alternative to estimate anaerobic power and capacity in one single test, with similar oxidative demand than AO30s.

## Introduction

Invasive metabolic parameters related to the anaerobic adenosine triphosphate (ATP) yield are difficult to measure. On the other hand, performance parameters are easier to determine, and have been used to quantitatively describe the function of glycolytic and phosphagen pathways [1–3], also known as anaerobic pathways (see Chamari and Padulo [4] for more details about the terminology). An all-out bout lasting 30 seconds (AO30s) is commonly used to evaluate fast ATP re-phosphorylation, since its high demand of ATP yield in a short period of time leads to high correlations between the mechanical power performed and phosphagen and glycolytic metabolic indicators [5,6].

Classically known as the Wingate test, performance parameters from an AO30s are often used to evaluate two distinct constructs called “anaerobic power” and “anaerobic capacity”, which represents the maximal rate and maximal amount of ATP resynthesis, respectively [7]. For that, Wingate test’s mechanical power, or the product between velocity and force produced during exercise, are retained for analysis as maximal and mean power. High correlations between AO30s maximal mechanical power and both phosphocreatine content and muscle acidosis level [6,8,9] support the use of this mechanical variable as anaerobic power estimator. Furthermore, correlations for mean mechanical power and anaerobic capacity [7, 10] reinforces the use of AO30s performance parameters to evaluate such metabolic function constructs in an easy, non-invasive way.

Although the practical advantages of AO30s to sports evaluation, during such exercise severe alterations in homeostasis are observed; among them are the accumulation of hydrogen ions (H+), inhibition of muscle glycogen phosphorylase, and even hypoglycaemia [11,12]. Therefore, developing maximal intensity during a 30 second bout could be a hard task, and the awareness of the discomfort caused by the break in homeostasis may result in restraint of the maximum performance regardless of external orientation and motivation, i.e. a sub maximal pacing strategy [10,13]. Such anticipation effect could reduce the validity of AO30s performance parameters as predictors of fast ATP metabolic pathways.

In comparison to shorter bouts, maximal mechanical power seems to be sub maximal in AO30s but not in a 20 s all-out effort (AO20s) [10,14,15]. Thus, using an effort with shorter duration may be more useful than the AO30s to evaluate the anaerobic performance. Furthermore, majority of results regarding this matter was obtained in a cycle ergometer, and since the modality of exercise is a key aspect on metabolic distress [16], the investigation on other types of exercise could help to find an optimum duration of exercise to evaluate anaerobic metabolism.

Additionally, few to none study objectively investigated the effort duration effect to metabolic and mechanical parameters in all-out conditions. Better understanding of the duration influence in short all-out efforts could be crucial to improve interventions such as SIT (sprint interval training) and other all-out based training to health and performance.

We hypothesized 30 s all-out bouts may not be the best duration to evaluate the combined function of glycolytic and phosphagen pathways. This study’s aims to verify the energy expenditure, metabolic distress and usefulness to evaluate the anaerobic constructs for different all-out durations in running efforts. Measures of oxygen consumption, excess post-exercise oxygen consumption (EPOC) and blood lactate concentration will be taken as metabolic function measurements and compared to tethered running performance in such all-out durations.

## Materials and methods

### Participants

Twelve active male (mean ± SD: age 21.0 ± 2.5 years (range 18–25 years); weight 79.6 ± 16.0 kg; height 178 ± 10 cm; body fat 16.8 ± 3.2%) took part in this investigation. Thirteen volunteers were initially recruited, but one was excluded for not showing to the last test session. They were enrolled in at least 30 minutes of physical activity 3–5 times a week, and were encouraged to keep food intake and hydration habits, as well as to avoid strenuous exercise 24h prior any testing session. All volunteers gave written consent to voluntarily take part in this investigation. The institution ethics committee previously approved all procedures before execution (5404 UNICAMP—campus Campinas), and all procedures complied with the declaration of Helsinki. CAAE number is 07116512.9.0000.5404 and can be consulted at: http://aplicacao.saude.gov.br/plataformabrasil/visao/publico/indexPublico.jsf.

### Experimental design

Four testing sessions were performed, interspaced by 24-48h. The first session consisted of anamnesis and familiarization with the non-motorized treadmill. An AO30s bout where performed in familiarization, with data not considered for analysis. Second to fourth test sessions had a randomized counter-balanced order, and consisted of standard warm-up (8km/h jogging followed by two ~2-3s maximum acceleration). After 5 min rest between warm-up and testing, volunteers performed one all-out effort lasting one of the following durations: 30 s, 20 s or 10 s. In all three test sessions volunteers received strong verbal encouragement to perform their maximal intensity through the test. At all moments volunteers were equipped with an portable gas analyser (K_4_b_2_, Cosmed, Italy) and blood samples were taken at rest, after warm-up, immediately post-test, and after 1, 3, 5, 7 and 9 minutes.

### Mechanical measurements

The bouts were performed on a non-motorized treadmill previously described [17, 18]. The participants ran attached by their waists by an inextensible steel cable in series with a load cell (CSL/ZL-250, MK Controle e Instrumentação Ltda, Brazil) for force measurement, and velocity was obtained as the first derivative of the treadmill displacement. Thus, power was the product between force and velocity. All signal measurements were performed at a high frequency (1000 Hz), and averaged for each second. System underwent daily calibrations.

Power is displayed as maximal (P_max_) and mean (P_mean_). Mean power was also calculated for the first 10 seconds (P_mean10s_) for normalized comparison between effort durations. Force and displacement curve integral was considered as mechanical work, calculated for the first 10s (W_10s_). To investigate mechanical power development over time, linear regressions (r^2^ between 0.89 and 0.97) were traced from the third second (where P_max_ occurred, in average) to the end, and slopes and y-intercepts were compared.

### Physiological variables

Oxygen consumption was measured and recorded breath-by-breath. Gas analyser was calibrated before each test session according to manufacturer recommendations for volume (3 L syringe), room air, breath delay and a certified determined gas mixture (16%O_2_, 5%CO_2_). Oxygen consumption data was smoothened using a low-pass digital filter (Butterworth, third order), with cut-off frequency of 4 Hz [19]. Peak oxygen consumption (VO_2peak_) was the highest value of the filtered data. Blood samples (25 μL) were drawn from the ear lobe using heparinized capillary tubes and stored in 400 μL 4% trichloracetic acid for posterior analysis of the blood lactate concentration. Blood lactate concentration was determined using a microplate reader according to Engel and Jones procedures [20]. All plates contained duplicate samples of reference lactate concentrations at 1, 2.5, 5, 10 μmol/L, and a first order regression equation was built between lactate concentration and absorbance for conversion of the samples in a given plate.

### Energy systems contributions determination

Total oxygen consumption of each test was obtained as the area under de curve of oxygen consumption over time, subtracted from the area equivalent to rest oxygen consumption, and took as the oxidative phosphorylation contribution to exercise. EPOC was obtained from the test end until 7 minutes post-test. A biexponential equation was fitted to obtain the EPOC_fast_, or the product between the first amplitude and coefficient of time [21]. EPOC_fast_ was adopted as the phosphagen pathway contribution. Peak blood lactate concentration ([lac]_blood_) was subtracted from [lac]_blood_ pre-test, to obtain net [lac]_blood_ and an O_2_-lactate equivalent of 3 mL O_2_ for each mmol·l^-1^ was used as the glycolytic pathway contribution.

### Statistical analysis

Descriptive statistics are reported as means ± standard deviation (SD). Data normality was checked (Kolmogorov-Smirnov test), and comparisons between effort duration for mechanical data and energetic contributions (absolute and relative) were performed via one-way ANOVA for repeated measures. When significant differences were encountered, Tukey-HSD test were performed as post-hoc analyses, and Cohen’s d were used to address the effect sizes (ES). ES were considered trivial (< 0.2), small (between 0.2 and 0.5), moderate (between 0.51 and 0.8) or large (> 0.8) [22]. In every case ANOVA or post-hoc analyses couldn’t reject the null hypothesis, intraclass correlation coefficients for absolute agreement (ICC-A) were performed to confirm agreement between variables (asterisk denotes ICC with p < 0.05). Relationships between mechanical data for different bout durations and mechanical data and energetic contributions in a same effort was performed using Pearson’s product moment coefficients. Significant differences in slopes of linear regression in power over time were tested via one-way ANOVA for repeated measures. Statistical significance was set at p ≤ 0.05 in all cases.

## Results

### Physiological variables

**Fig 1:**
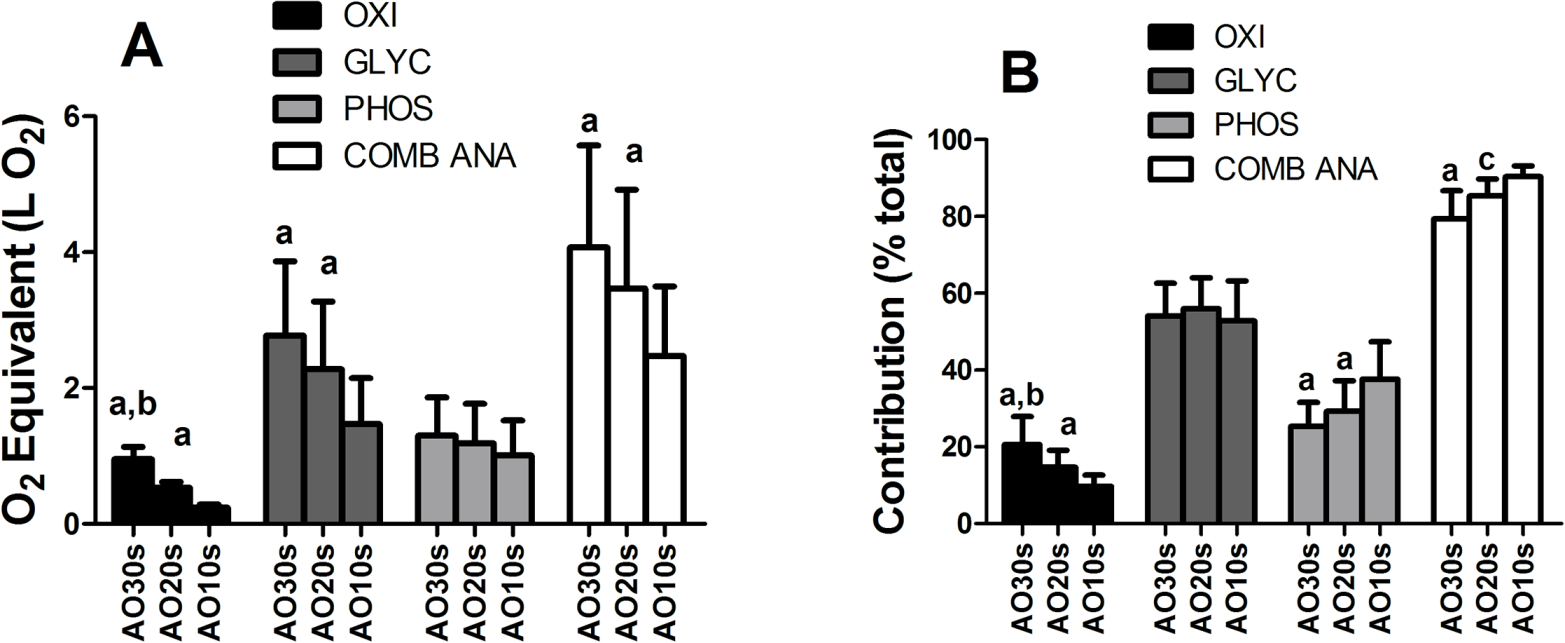
Energy expenditure in O_2_ equivalent (A) and relative to total (B), divided by metabolism. Detailed post-hoc results can be found on text. a–significant difference (p < 0.01) in paired comparison to AO10s; b–significant difference (p < 0.01) in paired comparison to AO20s; c–significant difference (p < 0.01) in paired comparison to AO30s.

Total (p < 0.001) and oxidative (p < 0.001) amount of energy expenditures were significantly higher for longer all-out durations, confirmed in paired comparison via post-hoc analyses (Fig 1A, p < 0.001; ES between 0.7 and 5.4). Glycolytic energy expenditures were also significantly different between bouts (p < 0.001), but post-hoc showed differences only between AO30s and AO10s (p < 0.001; ES = 1.4), and between AO20s and AO10s (p = 0.005; ES = 1.0). No differences could be reported between glycolytic energy expenditure in AO30s and AO20s (p = 0.11; ES = 0.5), with significant agreement confirmed via ICC (ICC-A = 0.63*). Phosphagen energy expenditures were similar between all-out durations (p = 0.12; ICC-A = 0.62*; ES < 0.5). Combined anaerobic energy expenditures were significantly different between bout durations (p < 0.001), but as glycolytic energy expenditure, post-hoc only reveal differences between AO30s and AO10s (p < 0.001; ES = 1.2) and AO20s and AO10s (p = 0.004; ES = 0.8), with no differences between AO30s and AO20s and agreement confirmed via ICC (p = 0.09; ICC-A = 0.75*; ES = 0.4).

A much higher oxidative relative contribution (p < 0.001; Fig 1B) could be observed for AO30s (20.6 ± 7.3%) than for AO20s (14.7 ± 4.4%) and AO10s (9.7 ± 2.9%), with post-hoc differences among all of them (p between 0.005 and 0.001; ES between 1 and 2). Glycolytic relative contributions were not significantly different between bout durations, but with agreement not confirmed by ICC-A (Fig 1B; ICC-A = 0.20). Phosphagen relative contribution performed a more important role in AO10s (37.6 ± 9.8%), being significantly higher than the other durations (vs AO20s: p = 0.05; ES = 0.9; and vs AO30s p = 0.004; ES = 1.5). Despite no significant differences for phosphagen contributions (p = 0.46; ES = 0.5) between AO20s (29.3 ± 7.9%) and AO30s (25.5 ± 6.2%), ICC did not confirm their agreement (ICC-A = -0.01). Considering combined anaerobic energy contribution, most of AO10s was anaerobic (90.3 ± 2.9%), having a significantly higher anaerobic contribution than both AO20s (85.3 ± 4.4%; p = 0.005; ES = 1.3) and AO30s (79.4 ± 7.3%; p < 0.001; ES = 2.0). Post-hoc analysis also revealed AO20s to have more relative anaerobic participation than AO30s (p = 0.001; ES = 1.0).

### Mechanical parameters

**Fig 2:**
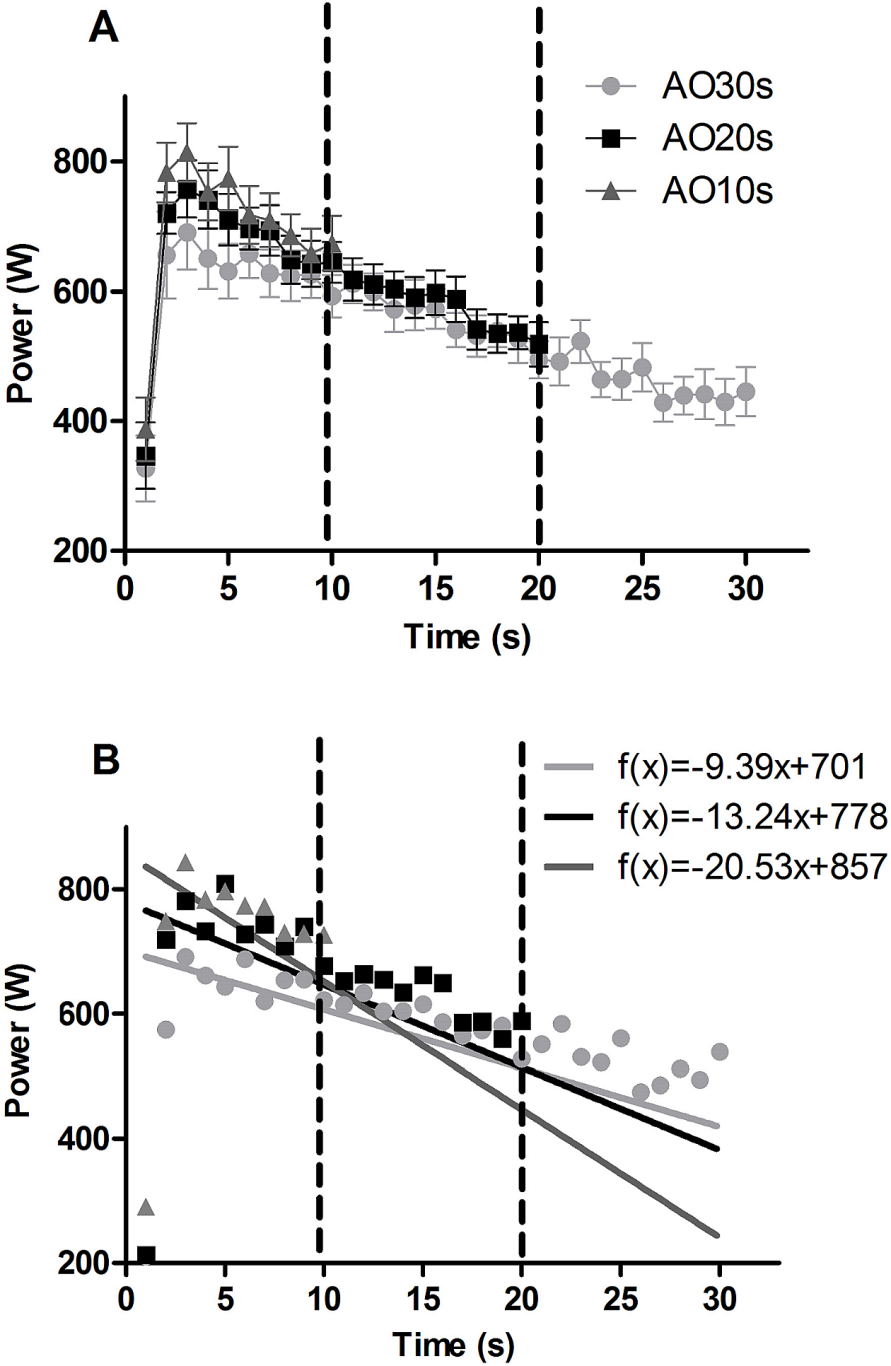
Power development averaged over 1s periods during the all-out bouts at the three effort durations. Results are displayed as mean and SEM (A) for all the volunteers. Continuous lines represent the regression analyses (B) considering the mean data.

Linear regression analyses presented in Fig 2 displays power development from the peak to the end of the test. All regression analyses presented a significant negative slope (p < 0.001), but were different between all bout durations (p = 0.02), being steeper for shorter durations.

All mechanical variables were significantly different between durations (Table1). Post-hoc analyses showed that P_max_ was higher in AO10s than in AO30s (p = 0.03; ES = 0.6), not being different between AO10s and AO20s (p = 0.67; ICC-A = 0.88*; ES = 0.2). As expected, P_mean_ were significantly higher for the shorter bouts (Table 1). When averaged using only the first 10 s, P_mean10s_ were still significantly different, but was significantly higher only for AO10s in comparison to AO30s (post hoc p = 0.027; ES = 0.7), with agreement between AO10s and AO20s (p = 0.23; ICC-A = 0.86*; ES = 0.3). Mechanical work performed at the first 10s of each bout were also different between bouts (Table 1), with post-hoc attesting higher values for AO10s than AO30s (p = 0.02; ES = 0.7), but not between AO10s and AO20s (p = 0.24; ES = 0.3). In addition, agreement for W_10s_ between AO10s and AO20s was confirmed via ICC (p = 0.50; ICC-A = 0.86*; ES = 0.3), but not for the other paired comparisons (p < 0.1; ICC < 0.45; ES > 0.5). Significant correlations were found between all different durations for P_max_ (r between 0.63 and 0.91; p < 0.05) and P_mean_ (r between 0.71–0.92; p < 0.05).

**Table 1:** Mechanical variables (MD ± SEM) for AO30s, AO20s and AO10s.

	AO30s	AO20s	AO10s	p-ANOVA
P_max_(W)	749 ± 167	813 ± 124	841 ± 155^a^	0.03
P_mean_ (W)	542 ± 101	617 ± 93^a^	696 ± 128^a^^,^^b^	<0.001
P_mean10s_ (W)	609 ± 136	660 ± 96	696 ± 128^a^	0.03
W_10s_ (kJ)	6.1 ± 1.4	6.6 ± 1.0	7.0±1.3^a^	0.03

post-hoc analysis

^a^–p < 0.05 in relation to 30s

^b^–p < 0.05 in relation to 20s

### Mechanical vs. physiological variables

During AO10s, P_max_ and P_mean_ were both significant correlated to total amount of energy expenditure, combined anaerobic energy expenditure and phosphagen energy expenditure (r between 0.72 and 0.75; P < 0.05), but not glycolytic and oxidative energy expenditure (r between 0.54 and 0.57; p > 0.05). Relative energy contributions did not present significant correlations to P_max_ and P_mean_. For longer efforts, less correlations between metabolic and mechanic variables were observed. For example, P_max_ was only significantly correlated with glycolytic energy expenditure in AO20s (r = 0.61; p = 0.03). In the other hand, P_mean_ was correlated to total energy expenditure and combined anaerobic energy expenditure in both AO20s and AO30s (r between 0.61 and 0.69; p < 0.05). Regarding relative energy contributions in AO20s and AO30s, P_mean_ was correlated to combined anaerobic contribution and inversely correlated to oxidative contribution in both AO20s (r = 0.65 and -0.65, respectively; p = 0.023) and AO30s (r = 0.58 and -0.58, respectively; p = 0.047).

## Discussion

This investigation aimed to verify the energy expenditure, metabolic distress, and usefulness to evaluate the anaerobic constructs for different all-out durations in running efforts. The hypothesis of 30 s not being the optimum duration to evaluate fast ATP re-synthesis pathways is supported by submaximal results of mechanical parameters (e.g. P_max_, P_mean10s_ and W_10s_) with similar phosphagen and glycolytic energy expenditure than the 20 s duration. Considering the results presented here, shorter bouts may be of better use for evaluation purposes and may require from “anaerobic” sources as much as AO30s.

Higher absolute and relative oxidative energy expenditure are verified for longer bouts, being nearly 21% of total energy expenditure for AO30s. This is in line with previous concerns, when Beneke, Pollmann [23] point out a substantial oxidative phosphorylation contribution in AO30s of about 18%. High participation of oxidative metabolism can be explained by time course of pyruvate dehydrogenase activity, since fifteen seconds is enough to fully activate this enzyme, with synchronic reduction in glycogen phosphorylase activity [10]. This is also in line with a significant oxygen consumption appearing from the middle of an AO30s to its end [24]. This may have happened because with the combined anaerobic pathways being heavily depleted, oxidative phosphorylation relative contribution increase in high intensity efforts [25,26]. This information is supported by Zagatto et al [27], where several 20 s efforts with progressive intensity interspaced by 100 s recovery periods until exhaustion presented a relatively high oxidative participation (65.4 ± 1.1%) even when not considered the recovery periods (26.5 ± 1.0%). The high relative contribution of oxidative phosphorylation pathway in AO30s shown here and in other studies may impair its performance variables as anaerobic fitness evaluators.

Despite the higher oxidative contribution in AO30s, glycolytic expenditure was not different between AO30s and AO20s, being lower only in AO10s. This suggests a similar glycolytic demand for AO30s and AO20s. Based on these results, prolongation of all-out effort from 20 to 30 seconds rely much on the oxidative metabolism. Since there is a possibility adaptations of a SIT protocol to be based on increased maximal glycolytic enzyme activity and/or Na^+^-K^+^-pump capacity [28], 20 s should be considered as an all-out duration capable of providing similar stimulus than 30 s to such ‘time efficient’ training intervention. On the other hand, based on the lower glycolytic activity presented here, 10 s may not elicit enough distress to be considered as a duration for a SIT protocol, explaining the results of previous studies using this duration in this type of training [29]. It is worth noticing only duration of effort was presented in the current study, these assumptions should be taken with care, considering the multifactorial nature of adaptations resulted of a SIT protocol, and the strong possibility of these adaptations to be more dependent on the oxidative distress caused by multiple bouts [16].

Current investigation results attests 10 s is enough time to elicit maximum phosphagen pathway energy expenditure, supported by the agreement between AO10s and longer durations. Since maximum rate of combined anaerobic energy expenditure happens in the first seconds of all-out exercise and was previously associated to phosphagen activity [9, 30], AO10s P_max_ may be a good alternative to evaluate maximal anaerobic power. Confirming this assumption, a study using a repeated sprint protocol (effort lasting ~5–6 s) P_max_ was only related to the phosphagen pathway activity (r = 0.65; p < 0.05), and not to the glycolytic (r = 0.28) and oxidative phosphorylation (r = -0.31) [26], similarly to what was found in the current investigation AO10s.

A significant steeper inclination in mechanical power (Fig 2) for AO10s than AO20s and AO30s proves that performance kinetics is different between effort duration, which needs to be taken into consideration when using performance parameters to evaluate energy expenditure from the phosphagen pathway. This explains P_max_ being significantly higher in AO10s than in AO30s, which is in accordance to Zajac, Jarzabek nad Waskiewicz [10] results. Same phenomenon could be observed for P_mean10s_ and W_10s_, with AO10s results being always higher than AO30s. In this investigation, the familiarization procedures included a AO30s to make sure all volunteers had previous experience with this kind of exercise. However, because of the difficulty and distress caused by an AO30s, it is possible that an unconscious pacing may have occur, as a self-preservation strategy. The volunteers were explained the need to not pace themselves, and strong verbal encouragement was given throughout the entire efforts in order to perform it in their maximum performance. P_max_ in ≤ 10 s durations was already shown to be higher than in AO30s [10, 15], but previous studies comparing 20 s to 30 s durations could not confirm these results since it divided the 30 s effort [31] or used double-blind designs [32]. If an unconscious pacing strategy happens in an AO30s, it could not be confirmed for AO20s in the current investigation, based in the comparisons to AO10s data. That said, for longitudinal monitoring of training, AO20s may be more suited since an unconscious pacing does not occur in this effort duration, at least for the population used in this study.

The correlation of total and combined anaerobic energy expenditure with P_med_ in both AO20s and AO30s suggests the usefulness of this parameters in both durations to estimate anaerobic capacity. Previous data shows correlation between P_med_ in AO30s and anaerobic capacity (r = 0.58; p < 0.05) measured by an adaptation of the maximal accumulated oxygen deficit [33]. Despite it has been discussed short all-out durations to not be enough to elicit maximum anaerobic capacity [34], previous data suggests that 30 s bouts seem to be long enough to use 80–90% of the maximum anaerobic capacity of a given subject [35].

### Practical applications

- AO20s return better performance parameters than AO30s to estimate anaerobic power and capacity;
- If the intention is to measure only anaerobic power, P_max_ from an AO10s is the best estimator among the studied effort durations.
- SIT and other high intensity training strategies should consider using AO20s as effort duration to confirm if it present similar results than AO30s;

## Conclusion

In summary, AO20s seems to be the better alternative between the studied effort durations to evaluate both anaerobic constructs, power and capacity. This way, 20 s is the best duration to evaluate non-mitochondrial pathways. Glycolytic demand in AO20s seems to be as large as in AO30s, making it a potential candidate for future studies using SIT and other all-out training strategies.

## Supporting information

S1 File Datasheet with all data used in the current manuscript. Folder “Physiologic variables” states the absolute and relative contributions from the different metabolic pathways. Folder “Mechanical variables” states P_max_, P_mean_, P_mean10s_, W_10s_ and displacement_10s_ for each volunteer and effort duration.
(XLSX)
